# Positive correlation between persistence of medical nutrition therapy and overall survival in patients with head and neck cancer

**DOI:** 10.3389/pore.2024.1611664

**Published:** 2024-03-15

**Authors:** Andrea Molnár, Erzsébet Pálfi, Barbara Belák, Célia Blasszauer, Dániel Reibl, József Lövey

**Affiliations:** ^1^ Health Sciences Division, Doctoral School of Semmelweis University, Budapest, Hungary; ^2^ Scientific Committee, National Association of Hungarian Dietitians, Budapest, Hungary; ^3^ Danone Hungary Kft., Budapest, Hungary; ^4^ Department of Dietetics and Nutritional Sciences, Semmelweis University, Budapest, Hungary; ^5^ Bacs-Kiskun County Hospital, Kecskemet, Hungary; ^6^ MedicalScan Kft., Budapest, Hungary; ^7^ National Tumour Biology Laboratory, National Institute of Oncology, Budapest, Hungary; ^8^ Department of Oncology, Semmelweis University, Budapest, Hungary

**Keywords:** medical nutrition therapy, survival, real -word evidence, persistence of nutrition and survival, head and neck cancer

## Abstract

**Background:** Several factors can affect overall survival of head and neck cancer (HNC) patients, including characteristics of the cancer disease and response to treatments. However, patients’ nutritional status and the effectiveness of medical nutrition therapy (MNT) can also impact overall survival. The primary goal of our research was to collect real-life data on the use of MNT in HNC patients and to specifically investigate the correlation between survival and the duration of uninterrupted (persistent) nutrition.

**Method:** The data of this retrospective, analytical, cohort study was collected from electronic healthcare records from the Hungarian National Health Insurance Fund Management. Overall, 38,675 HNC patients’ data of the period between 2012 and 2021 was used. We applied multi-step exclusions to identify patient groups accurately and to avoid biasing factors. Statistical analysis was done by the Kaplan-Meier method, log-rank test, and Cox regression analysis.

**Results:** Throughout the investigated period 16,871 (64%) patients received MNT therapy out of 26,253 newly diagnosed patients (≥18 years). In terms of the persistence of MNT, we divided the patients into three groups (1–3; 4–6; ≥7-month duration of MNT). When comparing these groups, we found that patients receiving long-term (≥7 months) MNT had a significantly longer overall survival (*p* < 0.0001) than those who received MNT for a shorter duration, both in locally advanced and recurrent/metastatic cases.

**Conclusion:** The main outcome of the study is that there is a positive correlation between the persistence of MNT and the overall survival in HNC patients when nutritional intervention lasts several months. It highlights the responsibility of the specialists during the patient journey to use MNT early and to continue its use for as long as it is beneficial to the patients.

## Introduction

Guidelines and clinical studies have shown that demographic characteristics (sex, age), cancer characteristics (stage, response to treatment), and nutritional status (presence of malnutrition, sarcopenia, cachexia and effectiveness of medical nutrition therapy (MNT) to treat them) can influence overall survival both independently and in relation to each other [1–7]. In the present article the authors address the issue of the effectiveness of MNT with a focus on the duration of uninterrupted MNT (referred to as *persistence*) and the survival in a head and neck cancer (HNC) patient population.

HNC is characterized by increasing incidence, prevalence, and still high mortality rates [8–12]. The 5- and 10-year survival rates are about 63% and 53%, respectively, and it is important to highlight that disease-specific deaths occur mainly in the first 2–3 years [8, 9]. One reason for the high mortality is that patients are often diagnosed at an advanced stage (due to diagnostic difficulties and lack of specific symptoms), and there is a high rate of local recurrence within 3 years (50%) [10, 13]*.* The more advanced the stage of HNC, the more invasive diagnostic and therapeutic procedures are required [14]. Treatment requires a multidisciplinary approach combining surgery, radiotherapy, and systemic therapy. These interventions on their own, and their combination even more, have short- and long-term consequences that have a significant impact on survival and other outcomes. While oncologists are looking for personalized therapeutic strategies to achieve more favorable outcomes, clinical expert guidelines highlight the need for early nutritional risk identification, early intervention (dietary advice, medical nutrition therapy when food intake is insufficient and escalation to tube feeding) [1, 2, 15].

The main aim of this study was to investigate the correlation between MNT persistence and overall survival. Another aim was to investigate the frequency and duration of uninterrupted MNT.

## Material and method

**FIGURE 1:**
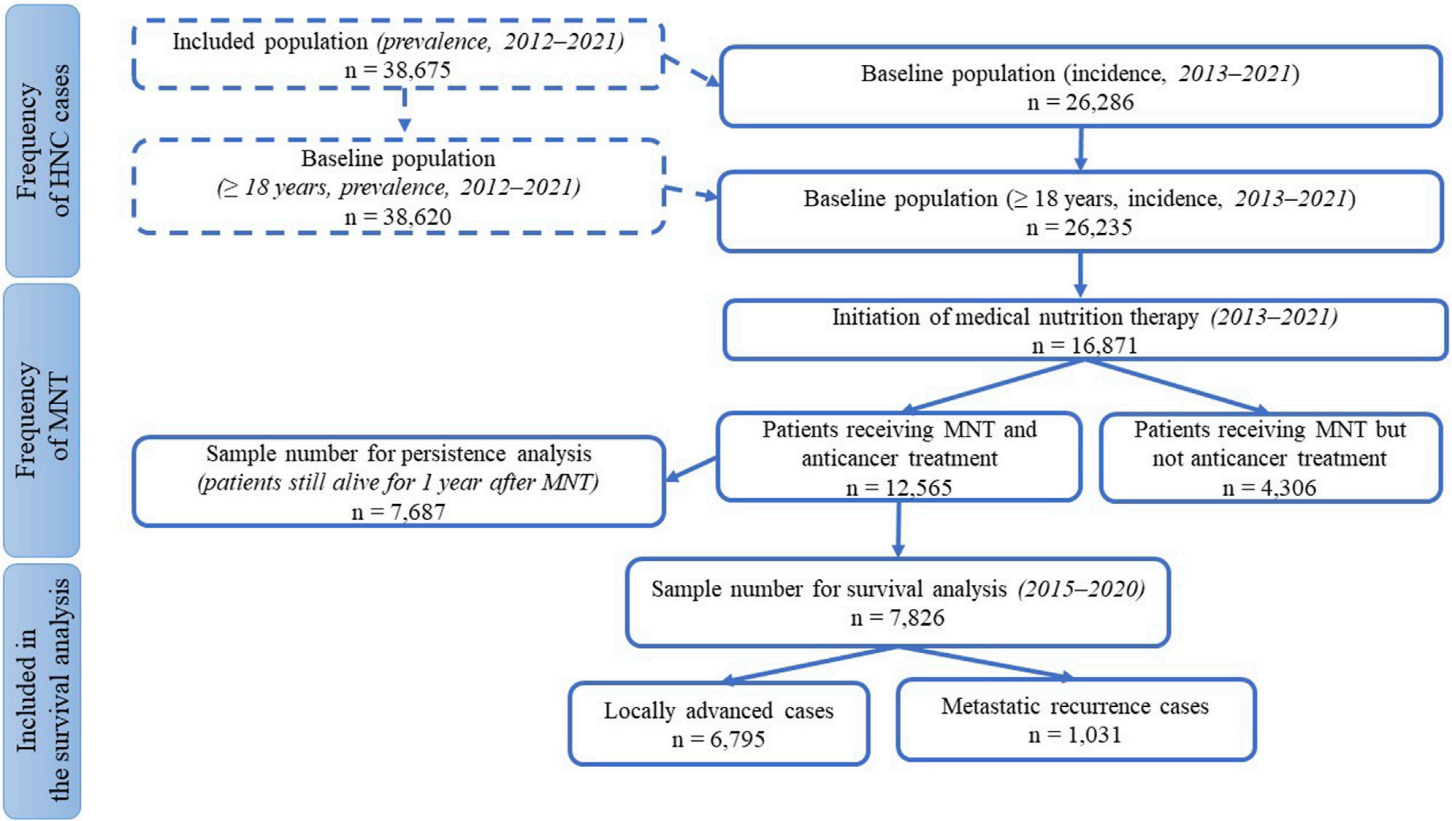
Patient numbers for subgroup analyses.

The study was a retrospective, analytical, cohort study of HNC patients using data collected from the Hungarian National Health Insurance Fund Management (HNHIFM) electronic health records. Ethical approval number is BM/4245-1/2023. For data collection and reliable conclusions, we needed statistical processing of a large data set on the frequency (prevalence, incidence) of Hungarian HNC patients, the type of treatments, as well as the use of MNT, its frequency, persistence, and method of administration. The total number of patients included in the study and the sample size available for each subgroup analysis are shown in Figure 1. A total of ten filtering criteria were developed to define the subgroup breakdowns. The term “medical nutrition therapy” was used to refer to nutrition therapy interventions when the physician ordered oral nutritional supplement (ONS) or enteral tube feeding (enteral nutrition).

For the frequency of HNC, we used three restrictive criteria for the inclusion: the International Classification of Diseases (ICD) code, the examined interval, and the adult age. From ICD we selected patients with codes C00–C14 and C30–C32 who had presented at least four times in inpatient or outpatient care (thus excluding presumptive but not confirmed diagnoses). We chose 10 years between 2012 and 2021 as the period of analysis (as baseline prevalence data), and of these, we considered 9 years of time for new patients (as baseline incidence data). We excluded patients aged under 18 years.

In terms of treatment, we collected in detail who had and who had not received anti-cancer treatment, as well as the type of treatment they had received.

Data on the use of MNT were obtained from the prescribing data for foods for special medical purposes administered orally (ONS) or enteral tube-feeding (enteral formula). In determining the frequency of MNT, the selection was based on the three screening criteria mentioned above (for new patients) with the inclusion of the patient receiving nutrition therapy as a 4^th^ criterion. For subgroup analyses on MNT persistence, we applied a 5th screening condition: we included only those who had also received antitumor therapy (thus eliminating the bias that could have been caused by shorter survival due to untreated status). In addition to the above, two further requirements were imposed: criterion 6th was that the interruption should not exceed 30 days to ensure continuity of MNT, and criterion 7th was that the patient should live for at least 1 year from the start of nutrition therapy. The latter criteria were necessary to exclude confounding factors that could rapidly shorten survival, such as very severe conditions that can no longer be controlled by anti-cancer treatment. We also investigated the relationship between MNT persistence and survival in the two main groups of patients receiving anti-cancer treatment: locally advanced (LA) and recurrent/metastatic (R/M). Here, at inclusion, only the first 6 of the above criteria had to be met by the sample, not the 7th, i.e., there was no expectation that the patient would live for 1 year after starting MNT. We thus expanded the number of patients, but further narrowed it by testing two additional criteria: as screening criterion 8th, we only examined data at a slightly shorter time interval (2015–2020) to achieve a more homogeneous cohort; as criterion 9th, we used the data of patients with a minimum survival of 90 days to exclude bias caused by short term mortality. For survival analysis, we divided patients into three groups based on a 10th criterion depending on the duration of MNT: short (1–3 months), medium (4–6 months), and long-term (≥7 months) continuous nutritional therapy. These lengths were chosen because the usual follow-up period of the patients according to the data of the HNHIFM was 3 months, and in Hungary the physicians can prescribe ONS and tube feeding formulae for 3 months. Furthermore, in relation to MNT, observational studies follow patients for varying lengths of time, with 3–6 months being common and longer studies being rare [16–18], so we also chose to create groups of patients on nutrition therapy for 3 months, 6 months, or longer. Survival was followed here for 365 days.

### Statistical analysis

The software “RStudio” was used to extract data from the HNHIFM database and to perform statistical analyses. Available data included socio-demographic data (e.g., age, gender), treatment and MNT information. Derived data were persistence and survival data. A *p*-value of 0.05 was set as the threshold for statistical significance (for Log-rank test and the other tests as well). The Kaplan-Meier method was used to estimate the probability of survival. Log-rank tests were used to assess the probability of mortality between groups. Cox regression model was used to evaluate the association between the survival time of patients and predictor variables, where the group receiving MNT for 1–3 months was the reference and compared with the group receiving MNT for 3–6 and ≥7 months.

## Results

### Summary of the results

Characteristics of the basic data used for the analyses (descriptive, categorization and distribution indicators): in Hungary, the prevalence of HNC is 38,675 patients for 10 years and the incidence is 26,286 patients for 9 years. The distribution of ICD codes based on prevalence data is presented in Table 1. Demographic characteristics of the study population is shown in Table 2. Data on treatment: 58.5% (15,380) of new patients received anti-cancer treatment and 41.5% (10,906) did not receive such treatment. Data on the frequency of MNT use: 64.3% (16,871) of new patients (≥18 years) received nutrition therapy between 2013 and 2021.

**TABLE 1:** The prevalence data of distribution of head and neck tumor diseases in Hungary between 2012 and 2021.

ICD codes	International classification of diseases (ICD), topographical codes	2012	2013	2014	2015	2016	2017	2018	2019	2020	2021	Summary
C00	Lip	217	273	256	273	265	279	236	294	237	239	**1,624**
C01	Base of tongue	702	737	794	764	751	720	704	703	642	604	**3,734**
C02	Other and unspecified parts of tongue	740	745	737	758	722	679	645	695	620	632	**3,749**
C03	Gum	140	149	132	128	146	154	146	149	127	165	**881**
C04	Floor of mouth	535	522	499	493	460	423	461	434	352	362	**2,430**
C05	Palate	275	272	293	282	266	238	278	274	188	203	**1,403**
C06	Other and unspecified parts of mouth	204	225	234	229	207	245	229	232	206	246	**1,397**
C07	Parotid gland	295	311	330	313	313	304	332	390	365	321	**1,775**
C08	Other and unspecified major salivary glands	103	122	139	145	133	122	125	158	116	137	**716**
C09	Tonsil	609	635	661	612	669	641	684	647	548	524	**3,056**
C10	Oropharynx	864	799	799	798	735	669	652	661	602	622	**4,054**
C11	Nasopharynx	277	287	285	299	283	289	283	294	236	240	**1,218**
C12	Pyriform sinus	103	130	123	117	109	96	106	94	81	82	**556**
C13	Hypopharynx	1,346	1,275	1,278	1,197	1,181	1,092	1,069	1,056	912	821	**5,952**
C14	Other and ill-defined sites in lip, oral cavity, and pharynx	783	834	876	889	884	978	867	887	835	777	**4,461**
C30	Nasal cavity and middle ear	120	117	115	125	137	128	140	147	144	151	**697**
C31	Accessory sinuses	155	155	167	146	163	134	136	141	123	129	**793**
C32	Larynx	3,225	3,229	3,225	3,171	3,028	2,917	2,838	2,752	2,384	2,378	**12,015**
Summary		**9,161**	**9,320**	**9,376**	**9,261**	**9,135**	**8,869**	**8,789**	**8,886**	**7,705**	**7,672**	**38,675**

Bold values indicate the local totals (subtotal values).

**TABLE 2:** Demographic characteristics of the study population.

Characteristic	Prevalence data		Incidence data	
	*N*	%	*N*	%
Sex				
Male	28,796	74.5	19,408	73.8
Female	9,879	25.5	6,878	26.2
Age (years)				
<18	55	0.1	51	0.2
18–65	21,816	56.4	16,149	61.4
>65	16,804	43.4	10,086	38.4

**FIGURE 2:**
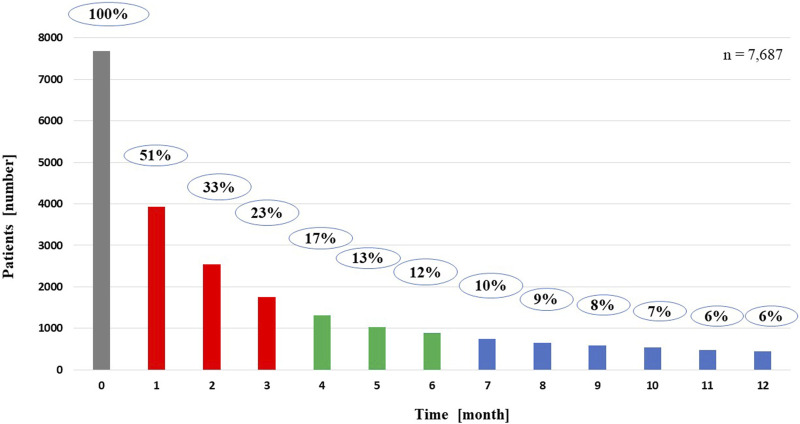
Persistence of medical nutrition therapy for patients living for 1 year from the start of nutrition therapy.

**FIGURE 3:**
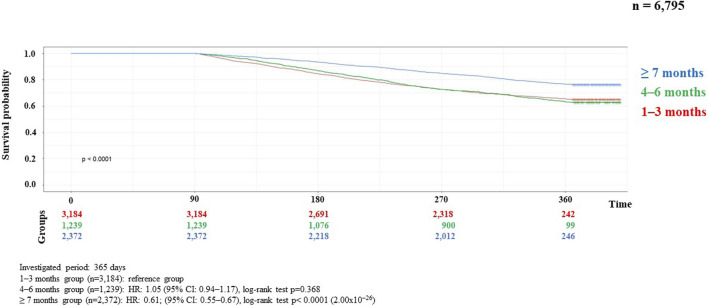
Kaplan-Meier curve of HNC survival probability in connection with persistence of MNT in locally advanced cases.

**FIGURE 4:**
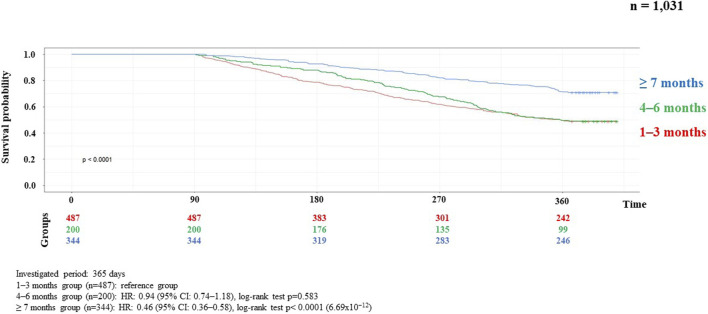
Kaplan-Meier curve of HNC survival probability in connection with persistence of MNT in metastatic recurrent cases.

Results of the correlation studies: After the examination of the persistence of MNT, the result of the relationship between the length of therapy and the decrease in the number of patients is presented in Figure 2, broken down by month and followed for 1 year. Initial number of patients: 7,687, followed by monthly decline: 3,928; 2,543; 1,762; 1,314; 1,027; 886; 744; 656; 584; 539; 482; 444. The main finding of the analyses examining the association between MNT duration and survival was that those receiving long-term (≥7 months) nutrition therapy had significantly better survival (*p* < 0.0001) than those receiving medium-term (4–6 months) (*p* < 0.368), compared to those receiving short-term (1–3 months) nutrition therapy. The results are presented in detail in Figures 3, 4.

### Evaluation of the results

To determine the prevalence of HNC, we compared our data with the Hungarian population at the beginning and the end of the period. In both cases, a significant decrease was observed, with prevalence decreasing from 94 to 79 and incidence from 35 to 25 per 100,000 population. The lowest numbers were seen during the COVID epidemic (2020–2021), but it is likely that the decrease was not due to fewer patients, but to a lack of diagnosis. In terms of demographics, we found almost equal proportions of sex in the prevalence and incidence data, with a higher proportion of under-18s and lower proportions of older people in the incidence data in terms of age. When evaluating the prevalence data by ICD codes, it can be said that most of HNC patients (31.1%, 12,015) had the code C32 (laryngeal tumor).

For anti-cancer treatment, the proportion of untreated patients was high (41.5%, 10,906), which is why it was necessary to exclude untreated patients when examining the impact of MNT on survival, as the significant number of untreated patients and their high mortality would have distorted the assessment of effectiveness. In the group of patients receiving anti-cancer treatment, 71.4% received treatment corresponding to locally advanced stage, 18.3% to the metastatic stage, and 10.3% received induction treatment.

Regarding the frequency of MNT use, the data were further analyzed according to whether patients had received anti-cancer therapy in addition to MNT, given the relatively high prevalence (64.3%, 16,871 patients) of MNT. In total, 74.5% (12,565 patients) received both treatments, 25.5% (4,306 patients) received MNT but not anticancer therapy. It is assumed that MNT was used as part of palliative treatment in the latter patients. A further observation is that the initial relatively high MNT rate is followed by a short therapeutic period, i.e., the proportion of patients on MNT drops to 51% by the first month, 33% by the second month, 23% by the third month, 17% by the fourth month, 13% by the fifth month, 12% by the sixth month, 10% by the seventh month, and then it further decreases until the 12th month, when only 6% of the patients received MNT. Figure 2 shows the rapid decrease in the number of patients on MNT in a monthly breakdown.

**FIGURE 5:**
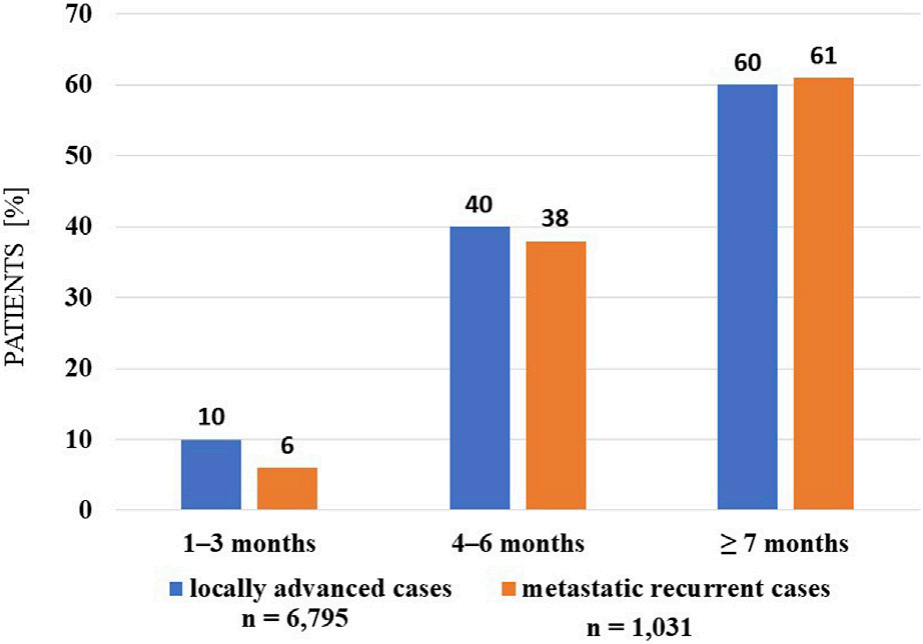
Percentage distribution of tube-fed patients in each therapeutic length group (investigated interval: 2015–2020).

Analyses of the persistence of MNT and survival showed that those receiving long-term (≥7 months) MNT had significantly better survival (both in the LA and R/M stage groups) compared to the group receiving MNT for 1–3 months (Figures 3, 4). There was no significant difference in expected survival for those receiving medium-term (4–6 months) nutrition therapy. Significant change (*p* < 0.0001) in survival in the figure was determined by the log-rank test and the null hypothesis that the survival probabilities are equal (i.e., there is no difference in survival probabilities among the groups), was rejected with 95% confidence. Based on the Cox regression calculation, we can state with 95% confidence that, compared to the MNT group of 1–3 months chosen as the reference, the 4–6 months group is not significantly different (*p* < 0.368), whereas the ≥7 months group is significantly different from the reference group (p < 2 × 10^−16^). One-year overall survival in the ≥7-month group was 76.3% for LA and 70.9% for R/M stage. Table 3 shows which specific treatments were used in the group breakdowns by the three treatment lengths. In further subgroup analyses we examined the number and the proportion of patients on tube feeding in both LA and R/M stage and found that their proportion was highest in the long-term (≥7 months) MNT group, 60% and 61%, respectively (Figure 5).

**TABLE 3:** Treatments cases depending on the length of Medical Nutrition Therapy.

Length of MNT [months]	Type of treatments	Locally advanced cases [number of patients]	Metastatic recurrent cases [number of patients]
1–3	Chemotherapy		372
1–3	Radiotherapy	2,926	
1–3	Radio-chemotherapy	473	
1–3	Immunotherapy		166
1–3	Cetuximab therapy		132
**1**–**3**	**Summary**	**3,184**	**487**
4–6	Chemotherapy		152
4–6	Radiotherapy	1,155	
4–6	Radio-chemotherapy	152	
4–6	Immunotherapy		74
4–6	Cetuximab therapy		56
**4**–**6**	**Summary**	**1,239**	**200**
≥7	Chemotherapy		256
≥7	Radiotherapy	2,238	
≥7	Radio-chemotherapy	304	
≥7	Immunotherapy		161
≥7	Cetuximab therapy		115
**≥7**	**Summary**	**2,372**	**344**

Bold values indicate the local totals (subtotal values).

## Discussion

Research based on real-world evidence (RWE) has received increasing attention over the past decade and plays a major role in healthcare decisions, including anti-cancer treatments and MNT [19, 20]. Provided that the quality of the data is robust and transparent, the results of RWE-based research are valuable complements to randomized controlled trials for evaluating treatment effectiveness.

The nutritional status and nutrient intake of HNC patients can vary depending on a number of controllable and uncontrollable factors, and it is the responsibility of the oncology care team to change the controllable factors in a positive direction, as far as possible [15, 21]. Timely screenings (malnutrition, sarcopenia, cachexia) help identify controllable, influencing factors, and nutrition counselling, then MNT is necessary to improve nutritional status (which means that improved nutritional status has a positive effect on the outcome of the disease and survival). Nutritional counseling is needed to help and to encourage patients to comply with their nutritional support requirement, at this time dietitians can give various suggestions, such as a high-energy and high-protein diet providing adequate macro-, and micronutrients. MNT is advised when the diet is not effective in reaching nutritional goals or if patients are unable to eat adequately (<50% of the requirement for >1 week or only 50%–75% of the requirement for >2 weeks). Enteral nutrition (EN) is recommended if oral nutrition remains inadequate despite ONS, and parenteral nutrition is recommended if EN is not sufficient or feasible [2]. Early introduction of MNT, including dietary counselling and the use of appropriate ONS with the proper daily dose and duration of therapy, are crucial for the effectiveness of the intervention [1, 2, 22, 23]. The majority of clinical research focus on early initiation and daily dosing, with length of therapy being a less researched area. In practice, there is clear evidence that the management of malnutrition in patients with chronic diseases is a continuous requirement along the patient journey and benefits of nutritional management are often only observed in the weeks and months following initiation. For cancer, no research has been done yet to investigate the relationship between the length of MNT and the expected survival in the particular types of cancer. However, for planning the duration of MNT, evidence is needed that shows, projected in months, the minimum intervention time that has a positive impact on survival.

Malnutrition is still under-diagnosed and under-treated in oncology patients, and international evidence-based research suggests that it is often prescribed as an end-of-life intervention or not used at all in oncology treatments [15, 20]*.* In a Hungarian study, Gallfy et al. also confirmed the above findings in a large sample population (1,616 patients) and found that 75.1% of patients would have required nutrition therapy intervention, but only 37.6% of patients received this intervention [24].

The uninterrupted duration of MNT is important in patients with cancer because achieving adequate nutritional intake is essential to support patients’ nutritional status that is often challenged due to increased protein breakdown and systemic inflammation induced by the tumor [1, 2, 25, 26]. Clinical guidelines generally recommend long-term nutrition therapy for the treatment of malnutrition associated with chronic disease, but do not address the length of MNT for each disease that has been shown to have an impact on outcome or survival, probably due to the lack of good quality data [22, 23, 27, 28].

The introduction and use of prehabilitation could also help achieve long-term MNT in patients with HNC. This would allow for the identification of patients at risk before surgery and other anti-cancer treatments and the early introduction of MNT, thus prolonging the time to therapy [29–32]. Prehabilitation could also allow for intervention before the signs of malnutrition are present and prevent deterioration in nutritional status. The cancer cachexia clinical practice guideline of ESMO recommends early prophylactic/preventative nutritional intervention approach in patients planned for anticancer treatment with a high risk of inducing malnutrition (e.g., combined-modality treatments, high-dose chemotherapy, highly emetogenic agents) [1].

The authors of the present article were interested in examining the above-mentioned facts in relation to real-life data on MNT for HNC patients in Hungary, with particular reference to the frequency of MNT and the duration of therapy. Analysis of the data from the study presented in this article showed that newly diagnosed patients received MNT in only 64% of cases, which seems very inadequate considering that 80% of patients experience significant unintentional weight-loss during treatment [26, 33, 34]. This rate is even worse when looking at the persistence of MNT. There are no disease-specific guidelines on the length of MNT that should be used for each disease, but if we consider active anti-cancer treatment as the minimum nutrition therapy time for HNC patients, then MNT for at least 3 months would be necessary! Based on the analysis of the correlations in our study, it is likely that a much longer MNT would be required for a better survival in both LA and R/M HNC stages. This can be easily explained by the exclusion of early mortality from our analysis. But when we look at survival in the shorter period MNT groups, we see that patients in R/M stage show worse outcomes than LA stage. Since this is not a direct comparison, we do not know if this is a real difference, but it suggests that MNT may play an even more important role in R/M cases. Although several plausible explanations could be put forward to support these observations, these are only hypotheses generated by the data and will need to be confirmed by further studies. Another interesting finding is that the proportion of tube-fed patients is highest in the long-term (≥7 months) groups, 60% in LA and 61% in R/M cases. It cannot be directly assumed that tube feeding leads to better survival, but once the tube device is already inserted, the patients are usually fed through it, resulting in a more favorable nutritional status compared to ONS consumption. Clinical experience has shown that patients sometimes give up consuming ONS due to, for example, the narrow selection of flavors, further reducing adherence to the therapy. Procedures that can be used in everyday practice to improve ONS adherence: regular monitoring and follow up in patients with an ONS prescription, reviewing patients’ acceptability and liking of the ONS they have been prescribed, and providing practical tips on how to incorporate ONS into daily life. In tube feeding adherence may be reduced by the extra care needed for the devices. In daily practice, it is observed that longer duration of MNT can be achieved with tube feeding, and this is confirmed by our research findings that the proportion of people receiving long-term nutrition therapy is higher than that of ONS users.

In conclusion, MNT is not used with sufficient frequency and duration of therapy in Hungarian HNC patients, and a correlation between long-term MNT and better overall survival was statistically demonstrated. In relation to these findings, we should mention the strengths and weaknesses of our data.

The obvious strength of our study is the robust sample size, which included a very large population with many patients. In Hungary, the most ONS and tube feeding formulas for MNT are reimbursed by HNHIFM. The data are highly accurate and reflect real evidence, as they include data on all care provided to patients, as well as on the dispensing of prescribed, ONS and tube feeding formulas, in pharmacies.

On the other hand, our study suffers from the usual drawbacks of retrospective population-based studies, such as cancer registry analysis. We treated large groups of patients, but had little information on in-depth details and changes, such as stages, histological subgroups, or comorbidities and side effects. In order to obtain realistic results, we had to apply a number of post-processing methods to filter out statistical noise, which are described in detail in the previous sections of this article. Although these subgroupings based on overlapping criteria helped analyze the data more efficiently, they also had the potential to introduce bias. Finally, it is important to note that retrospective analyses can never establish causality with certainty.

Because of the results that significantly affect the outcome of the disease, the initial results of the research were presented in the form of an English-language poster at the ESPEN congress in 2023, and due to the great interest, the authors published the contents of the poster in the form of a short article in a Hungarian Oncology journal, in the Magyar Onkológia [35].

## Conclusion

Our study has highlighted the fact that in Hungary head and neck cancer patients’ medical nutrition therapy is not optimal, neither in terms of the number of patients nor in terms of the persistence of the MNT. Even allowing for possible bias in the analysis, the data suggest that adequate and sustained MNT can improve overall survival. Therefore, it is recommended to review and optimize the current practice of nutrition therapy management of HNC patients in Hungary for better treatment outcomes and longer survival.

## Data Availability

The original contributions presented in the study are included in the article/supplementary material, further inquiries can be directed to the corresponding author.

## Abbreviations

CI, Confidence interval; EN, enteral nutrition; HNC, head and neck cancer; HNHIFM, Hungarian National Health Insurance Fund Management; HR, hazard ratio; ICD, international classification of diseases; LA, locally advanced; MNT, medical nutrition therapy; ONS, oral nutritional supplement; QoL, quality of life; RWE, real-world evidence; R/M, recurrent/metastatic; TNM, Classification of Malignant Tumors; T, tumor size; N, degree of spread to regional lymph nodes; M, presence of distant metastasis.
